# A soft, scalable and adaptable multi-contact cuff electrode for targeted peripheral nerve modulation

**DOI:** 10.1186/s42234-023-00137-y

**Published:** 2024-02-14

**Authors:** Valentina Paggi, Florian Fallegger, Ludovic Serex, Olivier Rizzo, Katia Galan, Alice Giannotti, Ivan Furfaro, Ciro Zinno, Fabio Bernini, Silvestro Micera, Stéphanie P. Lacour

**Affiliations:** 1 Laboratory for Soft Bioelectronic Interfaces, Neuro-X Institute, Ecole Polytechnique Fédérale de Lausanne (EPFL), Geneva, Switzerland; 2 Neurosoft Bioelectronics SA, Geneva, Switzerland; 3 Bertarelli Foundation Chair in Translational NeuroEngineering, Neuro-X Institute, École Polytechnique Fédérale de Lausanne (EPFL), Geneva, Switzerland; 4 The BioRobotics Institute and Department of Excellence in Robotics and AI, Scuola Superiore Sant’Anna, Pisa, Italy

**Keywords:** Peripheral nerve interfaces, Soft bioelectronics, Selective stimulation, Cuff electrode

## Abstract

**Background:**

Cuff electrodes target various nerves throughout the body, providing neuromodulation therapies for motor, sensory, or autonomic disorders. However, when using standard, thick silicone cuffs, fabricated in discrete circular sizes, complications may arise, namely cuff displacement or nerve compression, due to a poor adaptability to variable nerve shapes and sizes encountered in vivo. Improvements in cuff design, materials, closing mechanism and surgical approach are necessary to overcome these issues.

**Methods:**

In this work, we propose a microfabricated multi-channel silicone-based soft cuff electrode with a novel easy-to-implant and size-adaptable design and evaluate a number of essential features such as nerve-cuff contact, nerve compression, cuff locking stability, long-term integration and stimulation selectivity. We also compared performance to that of standard fixed-size cuffs.

**Results:**

The belt-like cuff made of 150 μm thick silicone membranes provides a stable and pressure-free conformal contact, independently of nerve size variability, combined with a straightforward implantation procedure. The adaptable design and use of soft materials lead to limited scarring and demyelination after 6-week implantation. In addition, multi-contact designs, ranging from 6 to 16 electrodes, allow for selective stimulation in models of rat and pig sciatic nerve, achieving targeted activation of up to 5 hindlimb muscles.

**Conclusion:**

These results suggest a promising alternative to classic fixed-diameter cuffs and may facilitate the adoption of soft, adaptable cuffs in clinical settings.

**Supplementary Information:**

The online version contains supplementary material available at 10.1186/s42234-023-00137-y.

## Introduction

Peripheral nerve interfaces are implantable neurotechnologies used to modulate or monitor the activity of peripheral nerves, for the treatment of motor, sensory, or autonomic disorders, as well as non-neurological conditions (Larson and Meng 2020). Extraneural cuff electrodes are widely considered the safest yet least selective among implantable nerve interfaces (Paggi et al. 2021). The design and development of cuff electrodes for targeting peripheral nerves primarily involves the creation of an extraneural implant which adequately surrounds the selected tissue. Designing an ideal cuff electrode presents several challenges, predominantly due to the wide variability in nerve targets in different animal models and in humans.

**Fig. 1:**
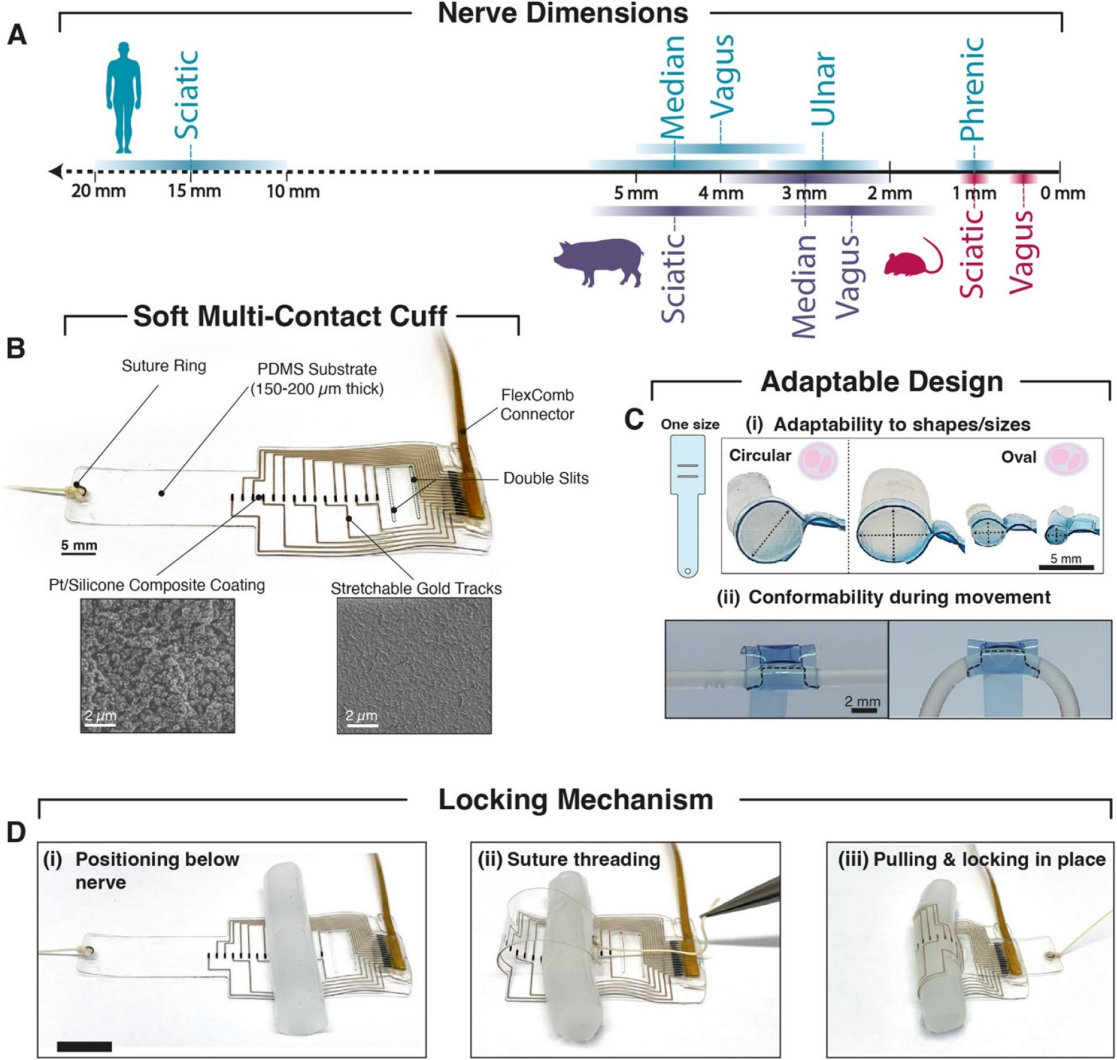
Easy-to-implant and adaptable soft cuff electrode. **A** Scale summarizing the range of nerve diameters of commonly targeted human, pig and rat nerves. **B** Example of fabricated soft cuff electrode with arrows indicating its components. Insets illustrate scanning electron microscope images of Pt-PDMS composite and microcracked gold film. **C** Schematic illustration of one cuff (left) which can adapt to different agarose phantom nerve sizes (2–7 mm) and shapes (i), and can conform (dashed line) during bending (ii). PDMS has been colored with blue coloring agent for increased visibility on phantom nerves. **D** Belt-like fastening method allows for easy surgical implantation: (i) pulling cuff belt below nerve (ii) passing suture through slits (iii) pulling belt through slits and locking in place. Scale bar 1 cm

Firstly, peripheral nerves vary significantly in size between different anatomical locations and species, making it difficult to develop a “one-size-fits-all” electrode (Fig. 1A). Furthermore, there is considerable variation in the size and morphology of the same nerve between different individuals or animals and along different nerve lengths (Hammer et al. 2015). Therefore, the unpredictability of nerve variability makes challenging to choose the appropriate cuff size, even when targeting the same in vivo model and nerve level. The fascicular organization of the nerve, a crucial aspect of nerve anatomy, presents additional challenges in cuff electrode design. Nerve fibers are arranged into bundles known as fascicles, each potentially serving different motor/sensory functions within the nerve itself (Stewart 2003; Badia et al. 2010; Jayaprakash et al. 2023). The fiber organization is not constant throughout the nerve, as nerve fibers merge, split, and reorganize along the nerve (Upadhye et al. 2022). Therefore, cuff placement and stability are critical to achieving consistent and optimal therapeutic effects. Beyond nerve size, there are significant inter- and intra-species differences in the number of fascicles and their organization, as well as the overall nerve shape (Pelot et al. 2020). These aspects further complicate the design process, as each factor influences the cuff’s geometry, electrode number, and distribution. A comprehensive understanding of these factors is therefore crucial in achieving the best possible design and functionality of cuff electrodes.

Since their development in the 1970s and 1980s (Stein et al. 1975; Naples et al. 1988), cuff electrodes have become a widely used tool in clinical and pre-clinical settings. These devices are the preferred choice to achieve a minimally invasive interface with the peripheral nervous system (PNS), with different designs aimed at simplifying the implantation procedure and ensuring a secure fit around the target nerve. However, standard cuffs often struggle to address the aforementioned challenges, associated with nerve variability, size, shape, and fascicular organization.

Commercial and clinical cuffs typically employ simple spiral, split-cylinder or helical designs (Stein et al. 1975; Naples et al. 1988; Nemeroff et al. 2006). While these designs have proven sufficiently effective in long-term implantations (Christie et al. 2017), they still have important limitations. In instances where nerve diameters are larger than nominal cuff values, the implant can exert external pressure on the nerve, leading to occlusion of blood flow and subsequent nerve degeneration (Cuoco and Durand 2000). Conversely, the cuff might be too loose, causing poor electrode contact and potential displacement along the nerve length (Eldabe et al. 2015). Additionally, the foreign body reaction (FBR) at the implant interface often poses a significant challenge to long-term implantation and functionality (Carnicer-Lombarte et al. 2021). Although the cuffs are fabricated using soft materials like silicone, the wall thickness employed (up to 1 mm) can exert significant pressure contributing to FBR (González-González et al. 2018).

Addressing these persistent problems, researchers are now exploring a new category of extraneural interfaces that leverage innovative materials and geometries. These novel devices aim to reduce mechanical mismatch at the interface (Lacour et al. 2016), improve conformability and increase the spatial resolution of stimulation. To this end, stretchable, polymeric cuff electrodes have shown promising results. For example, a soft cuff was created using a conductive viscoplastic polymer that can deform with the peripheral nerve (Liu et al. 2020). Others (Cuttaz et al. 2021) developed a fully polymeric cuff based on PDMS insulation and a PEDOT-PSS/PU composite as a conductive elastomer. These demonstrations have highlighted the benefits of using mechanically compliant materials to ensure an improved contact and reduced FBR, but multi-contact configurations, and connector integration are still pending challenges to be addressed. Indeed, these novel technologies are often validated in simple bipolar or tripolar ring designs, which have limited fascicular selectivity compared to multi-contact cuffs (Polasek et al. 2009). This is primarily due to limitations in interconnect resolution and the complex integration of high-channel count connectors and wires on these novel materials (Fallegger et al. 2020). To this end, a step forward was proposed in the form of a soft and stretchable multi-contact cuff using Au nanowires as conductors (Lienemann et al. 2023). While the feasibility of a soft and spatially selective technology was established, future work is needed to ensure ease of handling, a proper locking mechanism and the assessment of long-term stability.

In this work, we describe the development of a novel stretchable, selective, adaptable and stable cuff electrode for peripheral nerve interfacing. The work aims at answering the most pressing requirements in the field by focusing on material and mechanics, closing mechanism and its stability, number of electrodes, connector integration, biointegration, as well as electrochemical and in vivo performance.

Using a 150 μm silicone layer (E ∼ 1 MPa) as the base material, and incorporating stretchable thin-film gold tracks, the cuff was designed to adapt to a range of nerve sizes and shapes. It features a unique belt-like structure, ensuring near-complete perimeter coverage and facilitating implantation, while minimizing footprint and pressure on nerves to reduce FBR. Electrochemical characterization and rat sciatic nerve testing confirmed the device’s stable performance across varying conditions. The cuff’s applicability was further demonstrated in translational applications involving pig sciatic nerve stimulation with a 16-channel design selectively activating up to five independent muscles.

## Materials and methods

Detailed methods relating to device fabrication, phantom nerve fabrication and tissue processing are provided in the Supplementary materials.

### Cuff fabrication

The fabrication method is based on previously developed work combining conventional microfabrication techniques and soft lithography (Minev et al. 2015a; Schiavone et al. 2020; Fallegger et al. 2021) and is fully described and illustrated in the Supplementary material (Supplementary materials and Methods and Figs. S1-S2). Briefly, a 4″ silicon wafer is treated and coated with a dextran release layer, followed by spin-coating of 75 μm polydimethylsiloxane (PDMS). A 23 μm polyethylene terephthalate (PET) sheet is manually laminated on the PDMS, acting as a shadow mask. Electrode and track patterns are defined using a femtosecond laser (WS Turret, Optec, and thermal evaporation (Auto 306, Edwards) is used to deposit conductive Cr and Au (5/35 nm). An encapsulation layer is prepared on another silicon carrier by creating a PET/PDMS/PET stack (23/75/23 μm), with laser-patterned openings for electrodes and pads. The PDMS layer of the encapsulation is bonded to the substrate using oxygen plasma activation. The implant electrodes are coated with a platinum-silicone composite using screen-printing. Finally, a flexible PCB is aligned and connected to the pads, then covered with a silicone sealant as described previously (Fallegger et al. 2023). After curing, the outline of the implant is defined using laser cutting and finally the cuff is released by dissolving the dextran layer in water.

### Nerve coverage assessment

Image acquisition was performed using a digital microscope (Leica DVM6) on phantom polyacrylamide (PAAm) nerve cross-section surfaces with and without cuff enclosure. Quantification was carried out in ImageJ.

### Nerve compression quantification

Image acquisition was performed using a digital microscope (Leica DVM6) on phantom PAAm nerve cross-section surfaces with and without cuff enclosure. Quantification was carried out using Digital Image Correlation (DIC). Briefly, the sample was placed vertically below the camera, exposing the phantom nerve cross-section. Images were taken with and without cuffs. The strain map was then extracted using the open source Matlab (Mathworks) package NCorr (Blaber et al. 2015), which compared the strained image to the image at rest. The data was then extracted from the package and further analysed on Matlab 2019b.

### Locking stability testing

Cuff locking stability was measured through mechanical uniaxial pulling tests using a tensile tester (MTS Criterion Model 42, load cell 1 N) at a rate of 0.1 mm/s. Phantom nerves were glued and fixed to a needle, which was clamped to the tester. They were kept hydrated prior to testing. Maximal force before dislodgement was recorded.

### Electrochemical impedance spectroscopy

Electrochemical impedance spectroscopy (EIS) was performed in vitro (flat and locked cuff conditions) and in vivo. Measurements in vitro were carried out using a 3-electrode setup in Phosphate Buffered Saline (PBS) 1X with a platinum counter electrode and an Ag/AgCl reference. In vivo measurements were carried out in a 2-electrode setup, using a stainless steel needle, placed under the skin, as counter. EIS was performed using a potentiostat (REF 600, Gamry Instruments) sending 100 mV sinewaves from 1 MHz to 1 Hz.

### Voltage transient measurements

Voltage transients (VTs) were measured using an oscilloscope (Tektronix, MDO3014) connected to the working electrode and Pt counter electrode. Measurements were performed in PBS 1X, while stimulating in parallel with a current source (Isolated Pulse Stimulator Model 2100, AM Systems) with symmetrical biphasic cathodic-first pulses of 100 μA in amplitude, 300 μs in pulse width, and at a frequency of 100 Hz. In vivo measurements were carried out in a 2-electrode setup, using a stainless steel needle, placed under the skin, as counter.

### Rat cuff and EMG implantation

All animal procedures and experiments were approved by the Veterinarian Offices of the Canton of Geneva, Switzerland (License GE82). All surgical procedures were carried out under mixture of oxygen and isoflurane anesthesia with induction at 4% and maintenance at 2–2.5%.

For acute studies, a total of 5 adult female Lewis rats with initial weight of approximately 250 g were implanted with a soft cuff electrode on their left sciatic nerve, under isoflurane anaesthesia. The left hind paw was shaved prior to surgery. Briefly, the skin was incised at the mid-thigh level and separated from the underlying connective tissue by blunt dissection. The sciatic nerve was exposed by separating the fascia and dissecting deeper between the vastus lateralis and biceps muscles. The nerve was then dissected from the surrounding connective tissue over 15–20 mm, down to the point were it branched into tibial and peroneal components.

The soft cuff was carefully inserted and locked around the nerve in 2 different locations: proximal, as distant as possible from the branching, and distal, just above the nerve branching. Electromyography (EMG) bipolar needle electrodes were positioned through the skin targeting two antagonist muscles mediated through the sciatic nerve, the tibialis anterior (TA) and the gastrocnemius (GM).

Cuff-implanted rats were sacrificed immediately after surgery by an overdose of pentobarbital (Esconarkon, Streuli).

For chronic studies, a total of 2 female Lewis rats (initial weight approximately 250 g) were implanted with a soft cuff with thin FlexComb connector, while 2 others were implanted with control fixed-diameter cuffs purchased from CorTec (Micro Cuff Tunnel CorTec GmbH ø =1.2 mm). Both soft and control cuffs were glued with silicone (RTV 734, Dow Corning) to a 3D-printed backplug pedestal (Grey Resin, FormLabs). All surgical procedures were performed under aseptic conditions. Both electrode and pedestal were implanted similarly to previous work (Wurth et al. 2017). Two longitudinal incisions of 2 cm were made at the level of the mid-thigh and of the lower lumbar region (at L3-L5 level), respectively. Implantation hindlimb was alternated between left and right from one animal to another. A subcutaneous pocket was created between the thigh and back openings. Prior to implantation, cuff and pedestal were treated with UV-Ozone sterilization for 10 minutes. The 3D-printed pedestal was threaded below the pocket and stabilized via 4 sutures onto the fascia of the back muscles. The cuff was then inserted and locked in a distal position of the sciatic nerve as previously described. Connector and wires were positioned in a stress-release loop to minimize implant pulling during the leg movement. Skin was then sutured and closed around the incision sites. Control rats received an additional sham surgery (mid-thigh level skin incision, access to sciatic nerve, skin suturing) on the contralateral hindlimb. After surgery, rats were transferred to standard cages with food and water ad libitum and allowed to recover in a temperature- and light-controlled environment. Cuff implanted rats were sacrificed at the end of the experiment by an overdose of pentobarbital (Esconarkon, Streuli).

### Rat sciatic nerve stimulation, EMG recording and analysis

Electrophysiology measurements were performed in a faraday cage. The stimulation protocol was performed using a Multichannel Systems stimulator (STG4004). Biphasic, charge balanced, cathodic-first current pulses with a duration of 100 μs and pulse amplitude between 0 and 300 μA (steps of 20 μA) at 2 Hz (10 repetitions per current amplitude) were sent to the active sites in a bipolar configuration. Recording of EMG signals was performed through a differential AC amplifier (AM-Systems 1700) with a gain of 1000 and a band-pass filter between 300 and 5000 Hz, then denoised through a Hum Bug noise eliminator (50 Hz noise reduction). The data was successively acquired through an ADC (PowerLab, AD INSTRUMENTS) and processed offline in Matlab 2019b. The maximal response from both muscles was recorded in each experimental session and used to normalize all EMG signals. Windows of current amplitude which could enable distinct and selective muscle activation were identified. Specifically, selectivity for each of the two muscle was determined by calculating a selectivity index (SI). This was previously defined as the ratio between the normalized compound muscle action potential (CMAP) of a target muscle i, and the sum of the normalized CMAPs elicited in both the TA and GM muscles (Badia et al. 2011; Wurth et al. 2017):

$${SI}_i=\frac{CMAP_i}{\sum_{j=i}^n{CMAP}_j}$$

A threshold for selectivity was set when the SI for the considered muscle was higher than 0.6. Similarly to Badia et al., an additional constraint was added by only considering EMG responses above 30%.

### Pig cuff and EMG implantation

The protocol for the animal study (no. 278/2021- PR) was approved by the Italian Ministry of Health and was in accordance with the Italian law (D.lgs. 26/2014). Implantation was carried out similarly to previous work (Strauss et al. 2020). Briefly, one male farm pig (weight 30 kg) was included in this study (3–4 months old). The pig was premedicated with Zoletil. (10 mg/kg) and anesthetized using Propofol and maintained under 1–2% isoflurane in air enriched by 50% oxygen. The pig was then placed in a lateral position and target muscles were exposed by dissecting and removing the overlying skin between the knee and ankle. Seven bipolar intramuscular needles (Spesmedica) were implanted on seven muscles, which were identified through superficial stimulation with a hook electrode: gastrocnemius (GM), soleus (SOL), 5th extensor digitorum (EXT-D5), extensor digitorum longus (EXT-DL), peroneus tertius (PT), peroneus longus (PL), tibialis anterior (TA). The PT muscle was excluded due to poor implantation and excessive signal disturbance. Access to the right sciatic nerve was obtained by performing a 17 cm long incision between the patella of the left hindlimb and origin of the tail. A sternum spreader was inserted and opened to access the biceps femoris and gluteus superficialis. The connective tissue was removed to gain access to the sciatic nerve and its bifurcation. Two elastic bands were used to elevate the nerve. The soft cuff was locked first on the sciatic and later on the branching tibial nerve.

### Pig nerve stimulation, EMG recording and analysis

Bipolar stimulation was performed on 8 electrode pairs around the nerve circumference. Biphasic cathodic-first pulses of 10–400 μA (steps of 10 μA) with a pulse duration of 300 μs were generated by an IZ2 stimulator (Tucker-Davis Technologies) and transmitted to the selected cuff electrode pairs through a custom PCB. The EMG signals were acquired using a PZ5 NeuroDigitizer Amplifier (Tucker-Davis Technologies). Signals were amplified (× 1000) and bandpass filtered at 10–250 Hz, with an additional bandstop at 50 Hz. Data processing was performed in Matlab 2019b. Maximal EMG amplitude for each muscle was obtained considering all sessions (sciatic and tibial stimulation), while normalized amplitude and selectivity indexes were computed as previously described above, with EMG threshold in this case set to 0.4, similarly to previous work (Strauss et al. 2020).

## Results

### Microfabricated, stretchable and adaptable soft cuff

The proposed soft cuff electrode has been designed to allow for an easy implantation and adaptability to varying nerve sizes which can be found when targeting a certain nerve or animal species (Fig. 1A). The belt-like cuff (Fig. 1B) is based on the so-called “e-dura” technology (Minev et al. 2015a; Schiavone et al. 2020; Fallegger et al. 2021): it consists in a PDMS substate and encapsulation layer (accounting for a total thickness of 150 μm for use in small nerve diameters < 1.5 mm, and 200 μm for larger nerves), stretchable micro-cracked gold tracks and a blend of platinum particles within an elastomeric matrix at the electrode site, for improved mechanical and electrochemical performance (Minev et al. 2015b). The micro-crack structure allows the thin metallization to stretch by out-of-plane deformation while maintaining a percolating pathway which ensures electrical conductivity (Lacour et al. 2006). Previous reports on the selected technology (Schiavone et al. 2020; Fallegger et al. 2021) have highlighted the ease of customization and the high yield and reproducibility of the wafer-based process, therefore making it an optimal choice for fabricating implants for different types of nerves and electrode configurations. In addition, the selected technology can be considered suitable for applications in the highly dynamic nervous system, due to the minimal impact of strain on device functionality, as reported in previous work (2x impedance increase at 1 kHz with applied 45% strain) (Minev et al. 2015a). Electrical connection to external stimulation equipment is permitted by using a customized flexible printed circuit board (PCB), hereafter named “FlexComb” (Fallegger et al. 2023) or a microfabricated polyimide-based equivalent system (Supplementary materials and Methods) which is connected to the gold tracks through the same Pt-PDMS coating used at the electrode sites. The cuff is further comprised of a an adjustable fastener which is designed to pass across two slits, with the aid of a suture attached at the end of the belt. The design allows for a quick and easy locking mechanism (Fig. 1D), which does not require additional fixation aids such as suture or glue, and relies solely on the roughness and hydrophobicity of the silicone: the closure is guaranteed by the PDMS-PDMS contact between the belt and the slits and the PDMS-RTV contact of the belt and connector sealant. In addition, the low Young’s Modulus (~ 1 MPa) and reduced thickness allow for a conformal contact of a same cuff design to multiple nerve dimensions, a significant feature for clinical and animal nerve models with high inter- and intra-species nerve variation (Fig. 1C(i)). An additional benefit of the substrate choice is its conformability to small radii, which is desirable not only to allow an intimate contact with small nerves, but also to ensure maintained contact during bending (Fig. 1C(ii)).

### Nerve coverage and compression

**Fig. 2:**
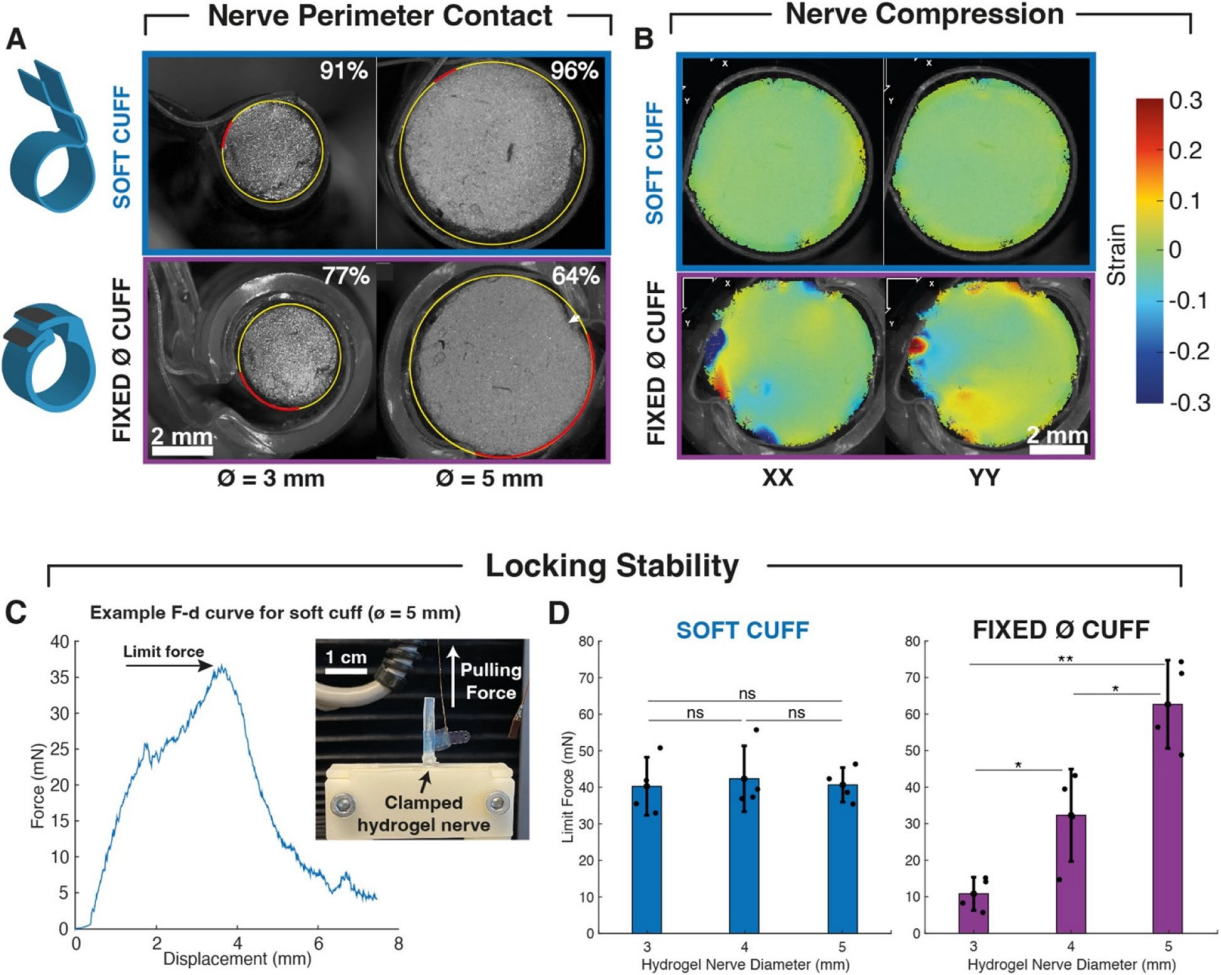
Cuff contact, compression and stability on phantom nerves. **A** Comparison of nerve perimeter contact of a soft and fixed-diameter cuff (schematically illustrated on the left). Quantification of soft cuff contact on 3 mm and 5 mm phantom nerve cross sections indicates full coverage, regardless of nerve size (top). Fixed cuffs do not ensure full contact (exposed areas in red). White arrow indicates visible compressive indentations (bottom). **B** DIC strain quantification of soft vs. fixed cuffs wrapped around hydrogel nerves of approximately 5 mm diameter. No area of critical compressive strain is visible with soft cuff. **C** Experimental setup of mechanical cuff pulling test (inset image) and a representative force-displacement curve with highlighted maximal force reached before cuff dislocation. **D** Maximal limit force for displacement of soft cuff (*n* = 4) indicates that stability is independent of nerve size, unlike fixed-diameter cuff (*n* = 4). Statistics: two-sample t-test, *p < 0.05, **p < 0.01, ns *p* > 0.1

The soft cuff can be designed to accommodate nerves of different sizes, with the ultimate minimum achievable diameter being determined by the presence of an area of enlarged PDMS width which cannot pass through the slits. Cuffs designed to lock around minimum diameters of 3 mm were tested on PAAm phantom nerves (3–4-5 mm) and compared to commercial cuffs, which are typically available in a range of few discrete circular sizes. The selected diameters were deemed appropriate to match the reported variability of the human cervical vagus nerve, with a nominal average value of 4 mm (Hammer et al. 2015). Quantification of digital microscope images (Fig. 2A) highlights the full perimeter coverage (over 90%) of the soft cuff in the case of both smaller or larger nerves compared to the nominal value, indicating a near-complete electrode-tissue contact also in the event of nerve size variability. Conversely, fixed-diameter cuffs do not ensure full coverage. DIC strain quantification in the X and Y directions on a 5 mm phantom nerve (Fig. 2B) was used to assess compression, which is known to lead to blood flow occlusion, resulting in nerve degeneration and axon demyelination (Powell and Myers 1986; Cuoco and Durand 2000). A decrease in local blood perfusion has been reported for sciatic nerves with compressive strains above 0.13 (Ju et al. 2006). The used PAAm yielded phantom nerves with an elastic modulus of 1.65 kPa, which is within range of reported mechanical properties of nerves (Paggi et al. 2021). No areas of the nerve phantom cross-section were found to be above critical strain thresholds when using soft cuffs, suggesting limited nerve damage upon implantation, while fixed-diameter cuffs lead to visible indentations and areas with local strain up to 0.3.

### Locking stability

Beyond potential nerve damage, poor cuff sizing could be detrimental for locking stability, with lead migration being a major cause of failure in peripheral nerve stimulation systems (Eldabe et al. 2015) and cuff “sliding” on target tissue leading to exacerbated FBR (Carnicer-Lombarte et al. 2021). In order to assess the stability of the soft cuff and its adaptability to different nerve sizes, a pulling test simulating the effect of tethering forces on the connector, was performed. PAAm nerves of 3–4-5 mm diameter were clamped and a pulling force was applied to the connector, while maximal force before displacement of the cuff was recorded (Fig. 2C). Soft cuffs and commercial cuffs exhibited different behaviour, with the soft cuff dislocation force being equal in all nerve configurations (Fig. 2D). Conversely, fixed-diameter cuffs did not allow for a tight locking when interfacing a 3 mm phantom and caused total fracture of the 5 mm nerve itself, suggesting a high risk of nerve compression when poor sizing is combined with large tethering forces, which can occur if strain relief loops are not properly implemented.

### Electrochemical characterization

**Fig. 3:**
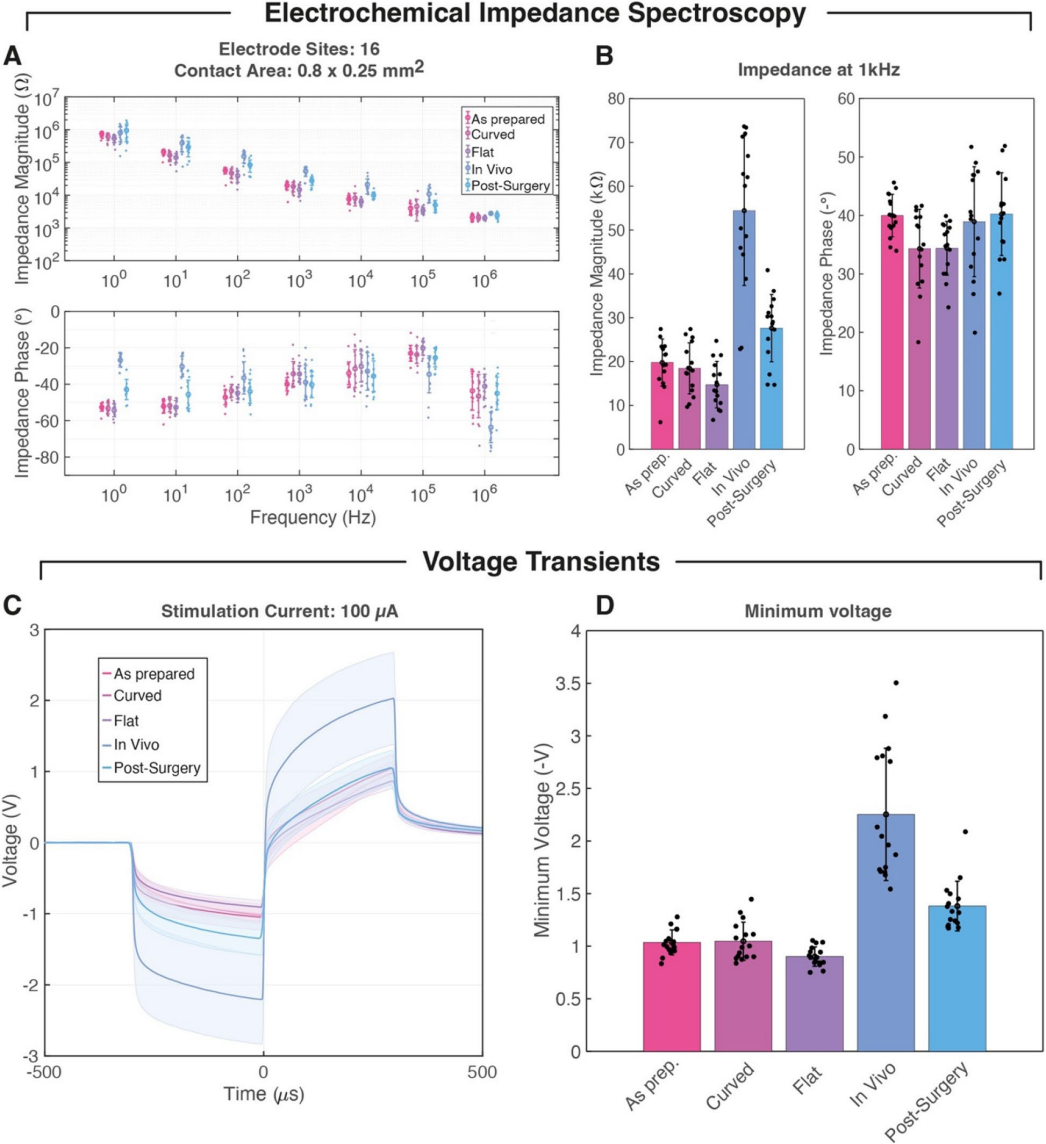
Electrochemical characterization of 16-channel soft cuffs. **A** Impedance spectra presented as mean ± std. dev (*n* = 16) at each decade, in 5 testing configurations. **B** Extracted impedance at 1 kHz in same testing conditions. **C** Average voltage measured on 16 electrodes (stimulation with 100 μA biphasic pulses) in 5 different conditions. **D** Extracted minimum voltage during stimulation for 16 channel soft Error bars (A,B and D) and shaded areas (C) represent standard deviation

Conformability and intimate electrode contact were further monitored by performing electrochemical testing. Soft cuffs were characterized through EIS and VT in different geometrical configurations. A soft cuff with 16 sites (A = 0.8 mm × 0.25 mm) designed for pig sciatic nerve was first tested in PBS flat, it was then locked around a PBS-agarose nerve of 4 mm, then tested flat again. Next, it was implanted (locked) in vivo in a pig sciatic nerve with approximate diameter of 4.5 mm. Lastly, the cuff was tested post-surgery in PBS in an unlocked configuration. EIS spectra (Fig. 3A) highlight no visible effect from curving in the 3 in vitro configurations, with magnitude and phase shifts appearing in vivo as expected due to the effect of the electrode-tissue interface. At 1 kHz (Fig. 3B) a 3x increase in magnitude can be attributed to the tissue and a minimal effect from the implantation was reported on the 16 electrodes post-surgery. Additional electrochemical characterization was performed by flowing symmetrical biphasic pulses at 100 μA through the electrodes while the voltage drop was recorded. Results in the different configurations and designs were consistent with EIS measurements, with similar increases in minimum voltage absolute value in vivo (Fig. 3C-D). The absence of an observable increase in voltage amplitude in the first 3 in vitro configurations indicates that the track resistance did not appear to be impacted by different folding conditions, while the net increase measured during intraoperatively is to be attributed to the effect of the electrode-tissue interface. Residual voltage increase after surgery may be attributed to tissue/blood residues on the electrode site.

Minimum voltage measurements were below 4 V in all cases at the set stimulation current, with most of the voltage drop being resistive due to the micro-cracked gold tracks. As implantable pulse generators (IPG) usually have a voltage compliance of 12 V (Zhang 2022), the electrodes could be used for currents up to 300–400 μA (electrode site: 0.8 × 0.25 mm^2^). In the following experimental sessions, current limits were therefore set to ensure safe and effective stimulation based on voltage transient measurements and known values of charge injection capacity and charge storage capacity of the Pt-PDMS composite electrodes, ∼ 60 μC/cm^2^ and ∼ 50 mC/cm^2^ respectively (Minev et al. 2015b).

### Selective stimulation of rat sciatic nerve

**Fig. 4:**
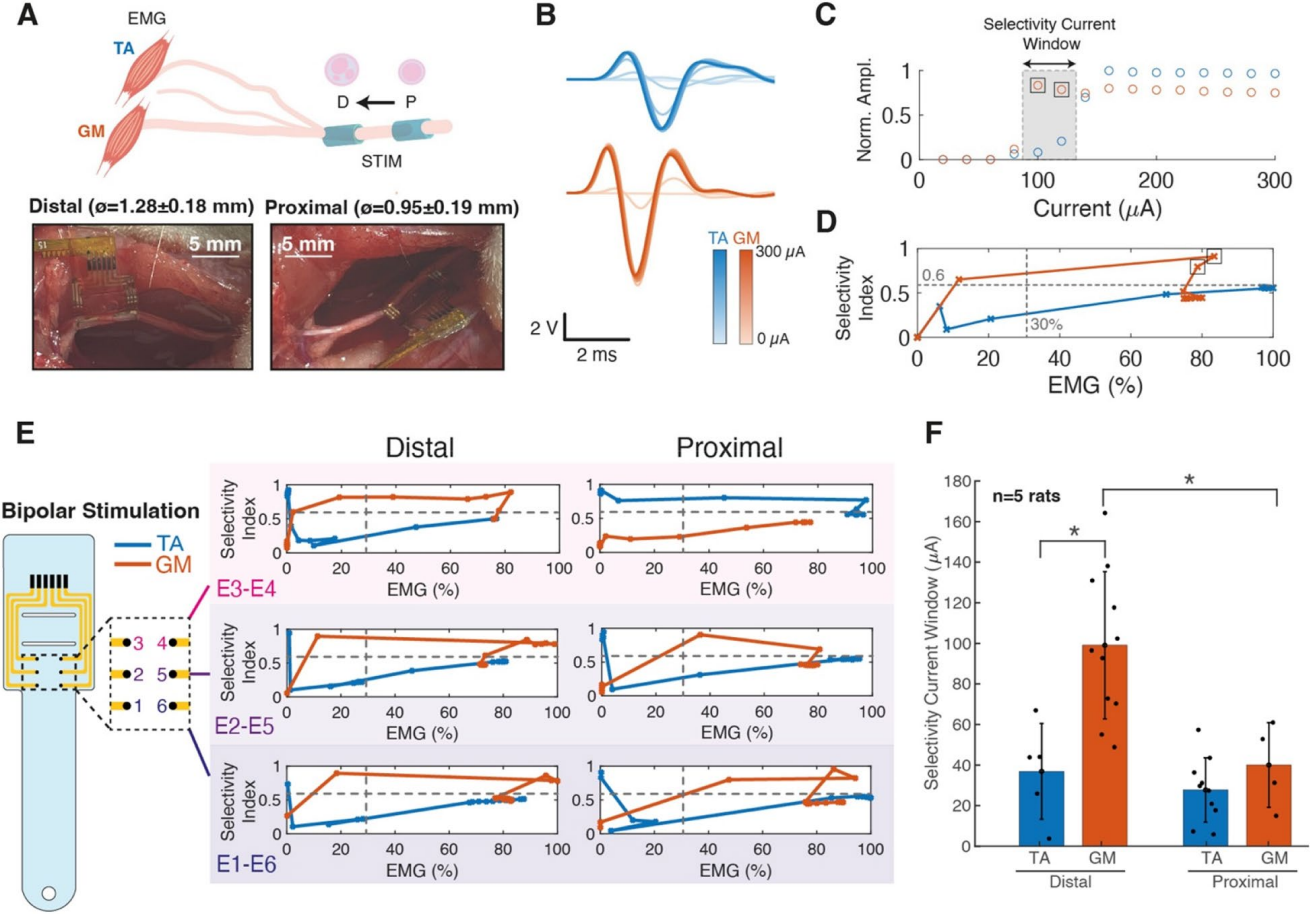
Acute rat sciatic nerve stimulation selectivity. **A** Schematic illustration of sciatic nerve stimulation experimental protocol carried out in both the distal and proximal nerve position, highlighting the anatomical differences between the different positions (top). Photographs (bottom) of soft cuff locked around a rat sciatic nerve distally (left) and proximally (right), with measured average dimensions of nerve locations. **B** Representative recruitment curves of EMG signals for TA and GM muscles with currents increasing from 0 to 300 μA. **C** Normalized EMG response as a function of current amplitude. Selectivity window indicates currents which preferentially activate one muscle. **D** Selectivity index as a function of EMG response. Dashed lines indicate 30% EMG response and 0.6 SI thresholds. **E** Schematic illustration of soft cuff with 6 channels used for bipolar stimulation and the corresponding selectivity achieved. Dashed lines indicate EMG response and SI thresholds. **F** Average selectivity current window in the distal proximal position for each muscle, with error bars denoting standard deviation. *p < 0.01, two-sample t-test (*n* = 5 rats). All data in B-C-D refer to Rat 1, E2-E5, distal position

The first in vivo validation of the soft cuff was performed using the rat sciatic nerve model, which has long been considered as one of the most suitable for the study of peripheral nerve injury and regeneration, due to its availability in pre-clinical research and its relative ease of manipulation (An et al. 2022). Cuffs were tested acutely in 2 positions along the rat sciatic nerve (*n* = 5), proximal and distal. In the distal position, electrode contacts were located 3 mm away from the nerve branching point, while the proximal location was on average selected at a distance of 6 mm along the nerve, away from the branching. In vivo quantification of rat sciatic nerves highlighted the known anatomical difference between proximal and distal nerve diameters, with the former measuring 0.95 ± 0.19 mm, and the latter 1.28 ± 0.18 mm (n = 5), with the soft cuff seamlessly adapting to both implantation sites (Fig. 4A). Increasing currents (0–300 μA) were applied to the cuff to elicit increasing EMG responses in both the TA and GM muscles (Fig. 4B), which are innervated respectively by the peroneal and tibial branch of the nerve. For each experimental session the maximal response was recorded and used to normalize EMG response and compute selectivity indexes (selectivity thresholds: SI > 0.6, EMG > 30%) and current windows (Fig. 4C-D), as described in the previous section. Specifically, in each session six electrode sites (ø = 250 μm) were activated in 3 bipolar configurations around the nerve perimeter and selectivity calculations confirmed the ability of the soft cuff to elicit independent muscle responses from certain electrode sites and configurations. As the rat sciatic nerve is composed of 2 main fascicles (peroneal and tibial) innervating distinct muscles, 3 bipolar sites were deemed sufficient to ensure activation of the different branches regardless of variations of electrode positioning from one experiment to another. A representative session (Fig. 4E) highlights the achieved selectivity for the TA muscle using the electrode pair E3-E4 in the proximal position, while all other configurations primarily activate the GM. Similar results were observed in all sessions, achieving independent activation of both muscles using different electrode pairs and cuff positions. The assessment of the average selectivity current window size in the different nerve locations underlines the accentuated selectivity towards the GM muscle when stimulating distally (Fig. 4F). This is likely to be attributed to the large size and defined structure of the tibial fascicle (innervating the GM) at the distal level, compared to the peroneal fascicle. Conversely, in the proximal position the fascicular organization is not clearly visible, which could therefore justify the similarity of muscle activations. In addition, stimulation properties were assessed, with activation thresholds always identified with applied currents in a range of 40–100 μA, independently of the position, and EMG plateau reached between 100 and 200 μA (Supplementary materials, Fig. S3).

### Foreign body reaction to chronic cuff implantation

**Fig. 5:**
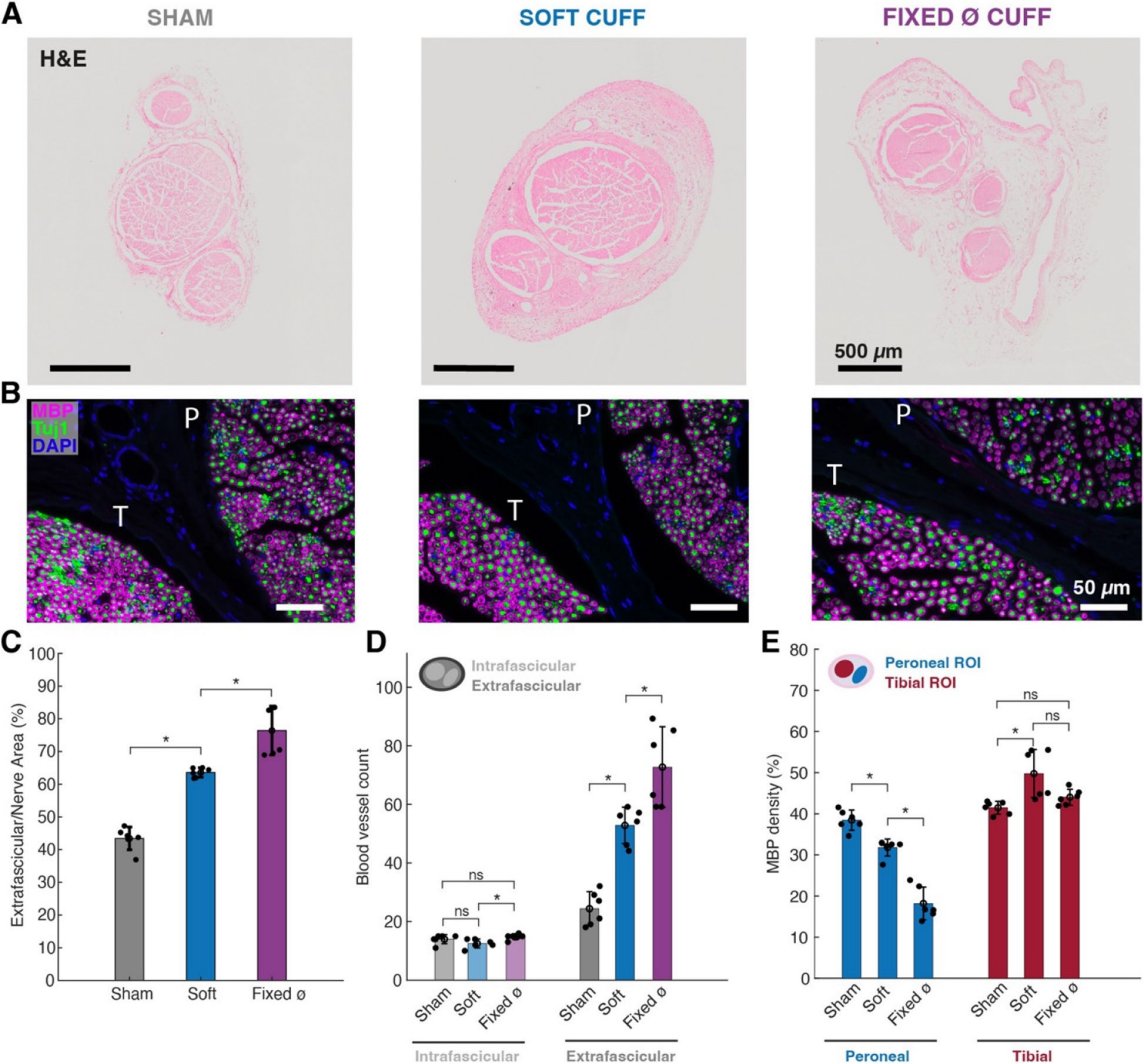
Foreign body reaction after 6-week implantation. **A** H&E slices for sham surgery (left), soft cuff (middle) and fixed cuff (right). **B** MBP, Tuj1 and DAPI staining of same implantation groups, illustrated in correspondence of both peroneal (P) and tibial (T) regions. **C** Proportion of total nerve area occupied by extrafascicular tissue. **D** Blood vessel count in different groups, highlighting increased vascularization in extrafascicular areas. **E** Quantification of myelin density in tibial and peroneal regions. Statistics: Wilcoxon rank sum test for * adjusted *p* < 0.05, with Bonferroni correction for multiple comparisons

Rats were implanted in a pilot study with non-functional passive soft cuff (*n* = 2) or a control commercial cuff with a fixed diameter (n = 2) to assess the effect of implantation on the sciatic nerve after 6 weeks. Additionally, control rats underwent a sham surgery on the contralateral paw. Different implantation groups could be identified by macroscopic morphological changes. Mainly, a thickening of the extrafascicular (epineural) tissue was observed after 6 weeks for both the soft and commercially-implanted rats (Fig. 5A), however a significantly smaller increase was measured for the soft cuff (Fig. 5C), indicating reduced nerve damage and compression. Similarly, an increase in vascularization in the extrafascicular space (Fig. 5D) was observed, correlating with the overall tissue increase, while endoneural vascular density did not appear to change. Indeed, the formation of fibrotic tissue in the extrafascicular space is typically accompanied by the formation of new blood vessels to support such growth and the metabolic needs of the tissue (Carnicer-Lombarte et al. 2021). Intuitively, a higher number of blood vessels is needed to support a larger area of newly-formed tissue Next, MBP and Tuj1-labelled slices were evaluated to quantify changes in axonal density and degree of myelination (Fig. 5B) which are classic signs of FBR due to implanted PNIs and nerve compression (Gupta 2004; Carnicer-Lombarte et al. 2021). The two main sciatic nerve fascicles (peroneal and tibial) were used as regions of interest (ROI) for the quantification. Interestingly, there was no observable effect on myelination at the whole-nerve level, whereas distinct effects could be observed in the separate peroneal and tibial regions (Fig. 5E). Specifically, cuff implantation caused a marked decrease in myelination in the peroneal fascicle, while it did not appear to have an effect in the tibial region, if not for a slight increase in the case of the soft cuff associated with one particular nerve. Similar, non-significant trends were observed in the case of axon density, quantified through the analysis of Tuj1 (Supplementary materials, Fig. S4A). A reduction in myelination and fiber density is to be expected, however the different observations in different ROIs could be indicative of a side effect of the implantation procedure and cuff location. Indeed, all four cuffs were implanted distally along the sciatic nerve, with the same medial-lateral orientation of the cuff. In particular, all devices were locked on the medial side, corresponding to the location of the tibial fascicle (Supplementary materials, Fig. S4B-C). As illustrated previously in Fig. 2A, the locking side is the one that leads to a partial coverage of the nerve, while the lateral/peroneal side should always remain covered. Additionally, all connectors and wires were threaded medially, therefore any cable pulling forces would be expected to cause more tightening on the opposite side. Furthermore, 3 out of 4 nerves exhibited a more noticeable tissue growth on the peroneal side, from H&E quantification, which can support the hypothesis of a localized damage linked to the implantation orientation and damaging mechanical forces caused by connector location and design (Larson and Meng 2020).

### Selective stimulation of pig sciatic nerve

**Fig. 6:**
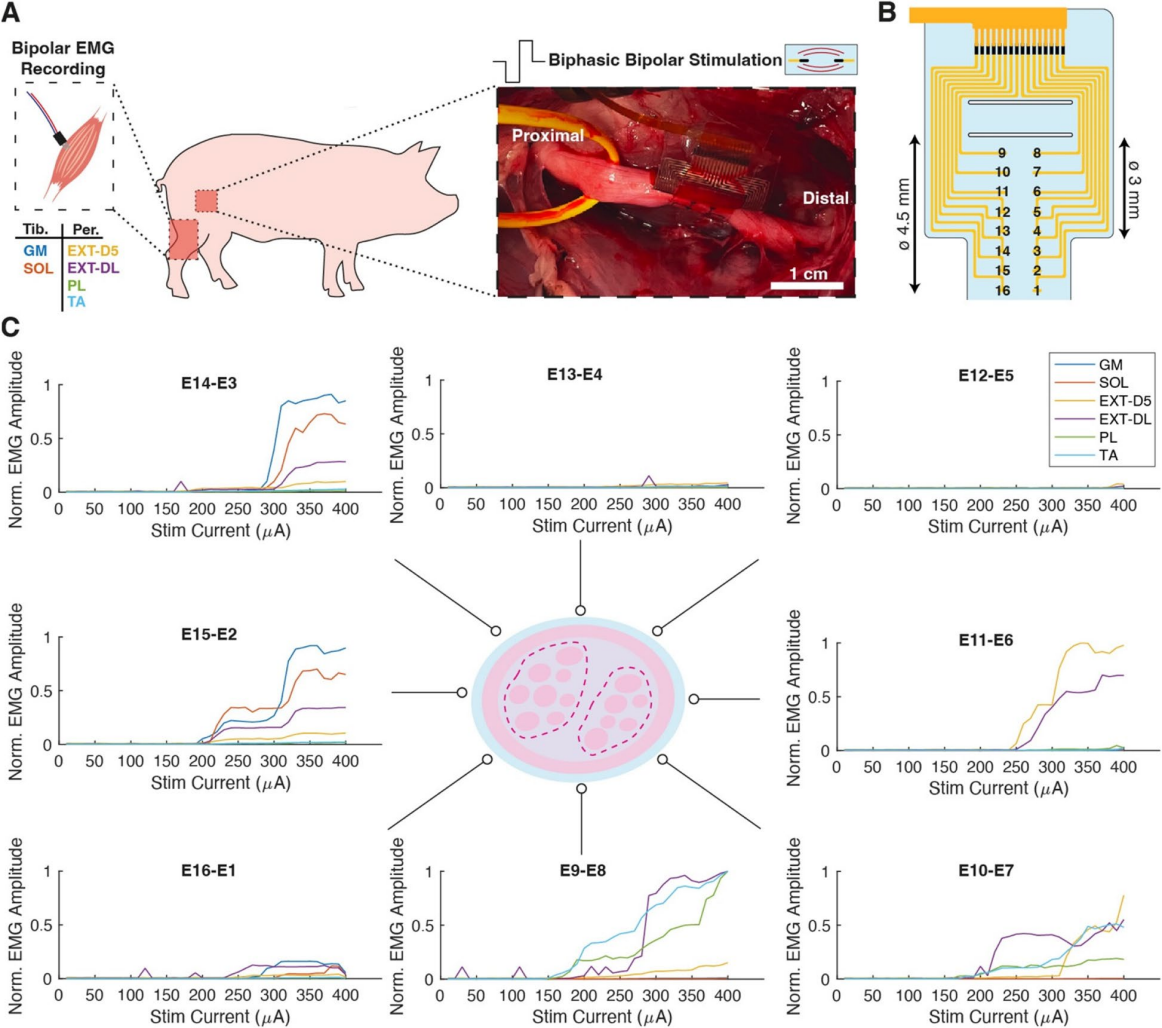
Selective stimulation of pig sciatic nerve. **A** Illustration of sciatic nerve stimulation and EMG recording experimental protocol. **B**) Schematic representation of cuff electrode sites. **C** Normalized EMG responses from each electrode pair located around the sciatic nerve. Nerve cartoon highlights the presence of a tibial (left) and peroneal region (right) within the sciatic

The soft cuff design was scaled and adapted to be employed in more clinically relevant models. Specifically, the pig sciatic nerve was chosen due to its similarities to the human nerve, in terms of size and complex fascicular organization (Server et al. 2018), to better assess the possibility of achieving fascicular selectivity. A 16-channel soft cuff was implanted acutely on a pig sciatic nerve of approximately ø = 4.5 mm and EMG recording was performed from 6 muscles innervated respectively by the tibial and peroneal branches (Tibial: GM and SOL, Peroneal: EXT-DL/D5, PL and TA), as illustrated in Fig. 6A. The cuff was designed to include 8 pairs of 0.8 mm × 0.25 mm electrodes arranged in a bipolar configuration (Fig. 6B), to perform biphasic bipolar stimulation up to 400 μA pulse amplitude. Full electrode functionality was assessed in vitro and confirmed in vivo, as previously illustrated in Fig. 3. As expected, different electrode pairs elicited responses from different muscles, grouped by innervation. A clear topographical organization can be identified and a reconstruction of sciatic nerve fascicular structure can be deduced (Fig. 6C). Specifically, three main categories of electrode activation were identified: sites primarily activating muscles with tibial innervation (E14-E3, E15-E2), sites activating muscles with peroneal innervation (E9-E8, E10-E7, E11-E6), and sites generating little or no responses (E13-E4, E12-E5, E16-E1), which we hypothesize to be located around areas constituted by the epineurium, which is made of connective tissue and does not contain nerve fibers. The soft cuff provided a conformal fit around the sciatic nerve, but could also be easily unlocked and implanted on the branching tibial nerve (ø = 3 mm), while still providing optimal contact (Supplementary materials, Fig. S5A). In this configuration, lower stimulation currents were required to activate and reach the plateau response for both the GM and SOL muscles (Supplementary materials, Fig. S5B), innervated by this branch. Maximal responses were recorded at 230 μA when stimulating in the tibial nerve, while responses from sciatic stimulation were elicited at 310 μA. On the sciatic nerve, 5 out of the 6 muscles could be activated selectively (GM, SOL, EXT-D5, EXT-DL), covering both tibial and peroneal muscle groups, and 5 out of 8 electrode configurations provided a selective stimulation (Supplementary materials, Fig. S5C). Fine movements, such as digit extension (sites E11-E6), could be achieved without eliciting overlapping large hindlimb movements. On the other hand, the tibial nerve implantation presented a higher number of non-selective and non-responsive sites. To some extent, this is to be expected as the last 2 electrode pairs (E16-E1, E15-E2) are not expected to be in contact as they exit the belt loop when the cuff is locked on smaller than nominal nerve diameters. The activation of the EXT-DL muscle is unanticipated as this muscle is generally reported as being mainly innervated by the peroneal branch (Strauss et al. 2020), however a secondary contribution to this muscle from the beginning of the tibial branch cannot be excluded and should be further explored with additional nerve mapping and EMG testing.

## Discussion

This work introduces a multichannel, soft and versatile cuff which addresses through its design the overlooked issues of adaptability, stability, nerve compression, and biointegration. The adaptability of the nerve interface was guaranteed by the ease of conformability of the thin PDMS substrate and the belt-like locking mechanism, which made it suitable for all nerve shapes and diameters encountered during all experimental session of rat sciatic nerve stimulation, a feature which could not possibly be achieved by fixed-diameter systems. The device also represents one of the first demonstrations of a soft and stretchable multi-channel cuff enabled by advances in microfabrication and packaging of soft materials, with small and large animal model designs ranging from 6 to 16 channels, a significant increase compared to simple 2-electrode configurations used in the clinic. The fundamental role of cuff positioning was demonstrated through stimulation experiments in different nerve locations, which highlighted the importance of finding and maintaining the optimal position to achieve selectivity, guaranteed by the multi-channel setup, and to ultimately ensure a better therapeutic outcome in clinical applications. Typically, the stability of nerve interfaces is obtained regardless of the design due to the formation of scar tissue, which locks the device in place. Ideally, the device should be stable without the need for additional scarring which acts as a barrier, distancing the electrodes from target tissues and causing stimulation thresholds to increase over time. The proposed cuff aimed at overcoming this problem by focusing on the importance of the locking mechanism and generating reduced scarring through the use of soft materials and small form factors for the both the cuff itself and the connector. Indeed, the cuff electrode was made of 150 to 200 μm-thick PDMS, with an elastic modulus of ∼1 MPa, values which ensured an ease of surgical handling, while also reducing nerve damage. A preliminary 6-week implantation study highlighted the potential for reduced nerve scarring and demyelination when using the proposed soft cuff, compared to a commercial fixed-diameter device. Results should be confirmed with a larger study at different time points, analyzing additional markers such as macrophages and fibroblasts to better evaluate the FBR and its evolution. In addition, the hypothesized effect of the cuff positioning and locking on myelin and axonal damage should be confirmed by testing different locking orientations. Further work should also address the functionality of the cuff in the long-term. A fully functional system should be tested chronically to verify how the stimulation thresholds evolve and if the selectivity is maintained thanks to the stable positioning.

Previous work with the same technology (Schiavone et al. 2020) has highlighted the implant stability during a 6-week implantation in the cervical epidural space of non-human primate spinal cord, through monitoring of impedance and voltage transients over time. In addition, long-term cycling experiments at 20% applied strain have reported minimal impact on electrode impedance magnitude (2x increase) after 1 M cycles (Minev et al. 2015a).

Such stability is promising also for this application in the PNS, while some deviations from the previous work can be expected due to the different dynamics between nerve and spinal cord, as well as different device design and electrode configurations.

The soft and stretchable cuff was successfully translated to large animal models with an application in pig sciatic nerve stimulation, demonstrating the scalability and translational potential of the design. The 16-channel cuff exhibited selectivity in up to 5 muscles, exceeding the performance of previous intraneural and stretchable multi-channel cuffs (Strauss et al. 2020; Lienemann et al. 2023). While the use of 8 bipolar arrangements of 16 electrodes has proven promising, further improvements could be achieved through the testing of different electrode configurations and stimulation schemes, optimized though the use of FEA modelling. As a starting point, similarly to previous work (Lienemann et al. 2023), 8 stimulation sites were deemed sufficient to obtain distinct activation of the main pig himdlimb muscle groups. Additionally, the number of active sites was also limited by the selected interconnect technology. In this configuration using micro-cracked gold technology, the minimum width of the interconnects is limited to 150 μm. Consequently, as the tracks are designed to travel along the sides of the central slits, the total width of the cuff can quickly become significant, especially when working with small animal models. Using another stretchable interconnect technology, such as kirigami-based approaches which allow track widths down to 30 μm (Vachicouras et al. 2019), would help minimize the total cuff footprint while maintaining a high density of electrode sites. Lastly, the stability and conformability of the cuff with growing or swelling tissue should also be addressed, similarly to previous work on growing nerves (Liu et al. 2020). Increase in nerve size is significant during development and growth (140% in Liu’s work) and would not be accommodated by the soft cuff design. The latter could however potentially be suitable for ensuring contact and functionality in case of pathological swelling in adult animals and patients (in neuropathy conditions, such as carpal tunnel syndrome). In these conditions, cross-sectional swelling can vary between 30 and 50% (Seok 2022), which corresponds to linear strain in the range of 15–25% and is expected to be tolerated by the stretchable technology, and to minimally compress the nerve. If suitable, the range of applications of the cuff design could be expanded to other tubular-like and expanding tissues, such as the gut.

## Conclusions

In this work, we have developed a soft cuff electrode for selective nerve stimulation. The belt-like structure of the stretchable silicone-based system allowed for an easy and conformable implantation for relevant nerve shapes and sizes, even during bending. The cuff can be designed to seamlessly interface with a selected range of diameters while always ensuring near complete nerve perimeter coverage. Simultaneously, the cuff can be locked in place without applying critical compression to nerves, even when these are larger than the nominal cuff diameter. The adaptability also ensures that its stability of positioning remains unaltered regardless of the nerve size, within the designed range. Electrochemical characterization further confirmed the achievement of an intimate electrode nerve contact, with the cuff performance remaining stable in different folding conditions in vitro, while intra-operative and post-surgery measurements confirmed the functionality of the device throughout acute experiments. Individual activation of muscles innervated by the sciatic nerve could be achieved in all experiments performed on rats, thanks to the multi-channel configuration, with the cuff adapting to both the small proximal location and the larger distal one. In pigs, we achieved selective activation of 5 out of 6 tested muscles. Finally, the preliminary biocompatibility study suggested a reduced growth of nerve tissue and minimal demyelination compared to the control fixed-diameter cuff, highlighting the importance of using soft and compliant materials in long-term implantations. In conclusion, the soft cuff design and materials are undoubtedly a promising solution for clinical exploitation, potentially simplifying the implantation procedure for surgeons, allowing an intimate contact with varying nerve sizes encountered in different patients, and achieving selectivity even in the case of complex fascicular organization, found in humans and large animal models alike.

### Supplementary Information

**Additional file 1.**

## Data Availability

The datasets used and/or analysed during the current study are available from the corresponding author on reasonable request.

## Abbreviations

EMG: Electromyography
FBR: Foreign Body Reaction
GM: Gastrocnemius muscle
H&E: Hematoxylin & Eosin
MBP: Myelin Basic Protein
PAAm: Polyacrylamide
PCB: Printed Circuit Board
PDMS: Polydimethylsiloxane
PET: Polyethylene terephthalate
TA: Tibialis Anterior muscle
