# Supplementary material for: A soft, scalable and adaptable multi-contact cuff electrode for targeted peripheral nerve modulation
Source: Bioelectron Med. 2024 Feb 14;10:6. doi: 10.1186/s42234-023-00137-y (PMC10865708; doi:10.1186/s42234-023-00137-y)
Supplement: Supplementary file 1 — Additional file 1. [file 42234_2023_137_MOESM1_ESM.pdf]

Supplementary materials

Manuscript Title

A Soft, Scalable and Adaptable Multi-Contact Cuff Electrode for Targeted Peripheral Nerve Modulation

Authors and affiliations

Valentina Paggi[1], Florian Fallegger[1], Ludovic Serex[2], Olivier Rizzo[1,3], Katia Galan[1], Alice Giannotti[4], Ivan Furfaro[1], Ciro Zinno[4], Fabio Bernini[4], Silvestro Micera[3,4], and Stéphanie P. Lacour[1,#]

[1] Laboratory for Soft Bioelectronic Interfaces, Neuro-X Institute, Ecole Polytechnique Fédérale de Lausanne (EPFL), Geneva, Switzerland

[2] Neurosoft Bioelectronics SA, Lausanne, Switzerland

[3] Bertarelli Foundation Chair in Translational NeuroEngineering, Neuro-X Institute, École Polytechnique Fédérale de Lausanne (EPFL), Lausanne, Switzerland

[4] The BioRobotics Institute and Department of Excellence in Robotics and AI, Scuola Superiore Sant'Anna, Pisa, Italy

# Corresponding author

Contents

Supplementary materials and methods

Figure S1

Figure S2

Figure S3

Figure S4

Figure S5

Supplementary materials and methods

Soft cuff fabrication

The general fabrication process of the soft cuff is depicted in figures S1 and S2 and is based on previously developed work (Fallegger et al. 2021) . A 4” silicon wafer was used as substrate carrier (fig. S1). The wafer was was oxygen plasma treated (100W, 0.2 mbar, 30s) and a 20%wt dextran solution in DI water (70kD, Arcos Organics) was spin-coated as a release layer at 2000 rpm, then dehydrated at 150 °C on a hotplate for 1min (i). PDMS (Sylgard 184, Dow Corning, ratio 10:1 base:curing agent) was spin-coated onto the wafer (660 rpm for 100 µm, 875 rpm for 75 µm) and cured at 75 °C for 2 hours (ii). A 23 µm PET sheet (Mylar, Lohmann Technologies) was then manually laminated onto the silicone, acting as a shadow mask for the thermal evaporation. To define the electrode and tracks a femtosecond laser is used (WS Turret, Optec) and the cut PET pieces were removed using tape (iii). In a thermal evaporator (Auto 306, Edwards) a 5 nm Cr adhesion layer was deposited and followed by 35 nm of Au (iv). Finally the stencil mask was removed (v).

Next an encapsulation layer was prepared (fig. S1B). First, a silicon wafer with a 25 µm PDMS layer was used as a carrier (i-ii). A PET sheet was manually laminated onto the silicone (iii) and then a new PDMS layer was spin-coated (75 or 100 µm) and cured as before (iv). The silicone surface was then covered with a new PET sheet (v). The PET/PDMS/PET was peeled off the carrier and laser cut with the femtosecond laser to define opening sites for electrodes and contact pads (vi).

The successive steps of substrate and encapsulation bonding and cuff assembly are depicted in figure S2. The top PET film was peeled off the stack and the PDMS/PET was bonded to the PDMS/Au substrate following oxygen plasma surface activation (i). The implant electrodes were coated with a platinum-silicone composite, as described in previous work (Minev et al. 2015; Fallegger et al. 2021). The composite was based on a 2:1 cyclohexane PDMS solution, mixed with Pt powder (0.27-0.47 um, Strem Chemicals), yielding a 30% wt PDMS to Pt composite. The paste was screen-printed to the electrode sites and pads by spreading over the second PET film, after an oxygen plasma activation.

A custom flexible PCB was aligned and placed in contact with the pads, the top PET film was then peeled off and the connection was sealed with silicone (RTV 734, Dow Corning) (ii). After an overnight reflow, the composite was cured for 4 hours at 55 °C.

Finally, the outline of the cuff was laser cut with the femtosecond laser (iii) and released (iv) by immersing the wafer in water, dissolving the dextran layer. An optional stainless steel ring was attached to the PDMS cuff using RTV silicone and a suture (4-0, Ethicon) was tied onto the ring as locking aid.

Dummy cuff fabrication

Passive cuffs of 150 to 200 µm thickness were prepared by spin-coating PDMS on a silicon wafer with a dextran release layer. Cuff outlines were laser cut and samples were released in water.

Control cuffs

Thick fixed-diameter cuffs were purchased from CorTec (Micro Cuff Tunnel CorTec GmbH, ∅ =4 mm, 25 µm PtIr metal sheet, ~ 500 µm thick silicone). These cuffs are hereafter labeled as 'fixed-diameter' or 'commercial', and they were used in comparative mechanical studies. Cuffs with 1.2 mm diameter from the same manufacturer were used in comparative chronic implantation studies in rats.

Fabrication of hydrogel phantom nerves

Two types of phantom nerves were fabricated with diameters of 3-4-5 mm. A 3% w/v Agarose (Agar Agar) to Phosphate Buffered Saline (PBS, 1X) solution was poured in tube moulds of desired diameters and kept at 4 °C. Phantom nerves were then manually extracted from tubes and used for imaging and electrochemical testing. Polyacrylamide (PAAm) based phantom nerves were prepared for mechanical tests. The preparation was based on existing protocols (Gao et al. 2019). Briefly, acrylamide powder was dissolved in deionized water (13% w/v), then N'-methylenebisacrylamide (MBAA, MBAA to acrylamide weight ratio 0.006:1) and APS (APS to acrylamideweight ratio 0.002:1) were added as crosslinker and initiator, respectively. The solution was injected into silicone tubes and heated at 75

°C for 1 hour to complete polymerization. After polymerization, dummy nerves were manually extracted from mould tubes.

Microfabrication of Thin FlexComb

A sacrificial layer of Ti/Al (20/100 nm) was deposited on a 4-inch silicon wafer by e-beam evaporation. A layer of SiOx/Ti (25/5 nm) was sputtered (AC450, Alliance Concept) following Ar-based surface activation. Next, an 8 µm-thick layer of polyimide (PI2611, HDMicrosystems GmbH) was spin-coated on the wafer and cured for 2 hours at 300 °C in a N_2 oven for hard bake. A conductive film of Ti/Pt/Ti (25/300/25 nm) was sputtered following O_2 plasma activation, and patterned through photolithography (ECI 3027,MicroChemicals, GER) and Cl_2-based Reactive Ion Etching (RIE, Corial 210L). After an O_2 plasma activation at 200 W for 30 s, a second 8 µm-thick layer of polyimide was spin-coated and cured to encapsulate the tracks and a top layer of Ti/SiOx (5/25 nm) was sputtered following Ar-based surface activation. A second photolithography was performed using an 18 µm photoresist (AZ10XT, MicroChemicals, double coating), followed by a succession of CHF_3, O_2 and Cl_2-based RIE to shape the connector, expose the pads and remove the top Ti. Finally, the individual devices were released through the anodic dissolution of aluminum in a 1.5 V bias in saturated NaCl solution. Released FlexComb connectors were integrated to cuffs as previously described. Connectors were O_2 plasma activated and placed in polymethyl methacrylate (PMMA) moulds filled with PDMS (10:1 ratio base: curing agent). Silicone overmoulding was cured for 2 hours at 75 °C and finally the connectors were manually demoulded from the PMMA.

Perfusion and tissue handling

After 6 weeks of cuff implantation, rats were sacrificed with an overdose of pentobarbital and perfused transcardially with a 0.1M PBS preparation containing 1% v/v heparin, followed by a perfusion with 2% paraformaldehyde (PFA) and 0.5% glutaraldehyde (Electron Microscopy Sciences, 25%). Both the implanted sciatic nerve and contralateral nerve were harvested and fixed overnight in the same PFA solution at 4 °C. After postfixing, nerves were transferred to 0.1MPBS azide (0.03% NaN_3) for long-termstorage at 4 °C. Next, nerves were embedded in HistoGel (Histocom). Each nerve was then

processed through routine cycles of graded alcohol, xylene and paraffin infiltration (VIP6, Tissue-Tek). Processed nerves were embedded in paraffin blocks (Canova embedding module, Diapath), then sliced transversally at the level of the implant in 4 µm-thick slices using a microtome (HM 355S, Microm) and mounted on glass slides (Superfrost Ultra Plus, Thermo Fisher Scientific). Slides were kept at 37 °C overnight and then stored at room temperature until further processing.

Histology

Hematoxylin and Eosin (H&E) staining and coverslipping was automatically performed with a LEICA ST5020 multistainer. Immunofluorescence was performed by first carrying out a dewaxing step with successive cycles of xylene and ethanol, a 0.1M PBS rinse, followed by an antigen retrieval process: slides were covered with citrate buffer for 10 min, followed by a succession 0.1M PBS rinses. Next, non-specific binding sites were blocked by incubating slides in 1% bovine serum albumin in 0.1M PBS for 1 hour at room temperature (RT). Slices were then processed for staining against myelin (myelin basic protein MBP, 1:200, Abcam) and axons (anti-beta III tubulin TUJ1, 1:200 Abcam) with an overnight incubation at 4 °C. After a washing step in 0.1M PBS, a 2 hour RT incubation was performed using the following secondary antibodies in 0.1MPBS: goat anti-mouse Alexa 488 (Life technologies A11001, 1:500), donkey anti-rabbit Alexa 647 (Life technologies A31573, 1:500). After a washing step, the tissue was counter stained with DAPI (1 mM, Cayman Chemical 14285) applied for 15 minutes, and a last PBS wash was performed prior to coverslipping with ProLong antifade reagent (Thermo Fisher Scientific).

Image acquisition and processing

Images of H&E slices were acquired with an optical microscope at 20x magnification (Olympus slide scanner VS120-L100, Olympus Corp.) while immunofluorescence stained tissue were imaged at 40x. Determination of cross-sectional area of total nerve and fascicles, as well as blood vessel count were carried out on H&E sections using QuPath software version 0.4.3 (Bankhead et al. 2017). Degree of myelination and fiber density were determined as the ratio of total stained area over the region of

interest (ROI), and then quantified with custom trained classifiers and intensity thresholding in QuPath, respectively.

Statistics

Quantification was performed on 3 slices per nerve. Non-significance of confounding effects (implantation side, rat ID) were determined using the Kruskal Wallis test. Pairwise comparisons between sham, soft and commercial cuff were performed with Wilcoxon rank sum test and significance was considered for p values below 0.05, with applied Bonferroni correction for multiple comparisons.

Supplementary figures

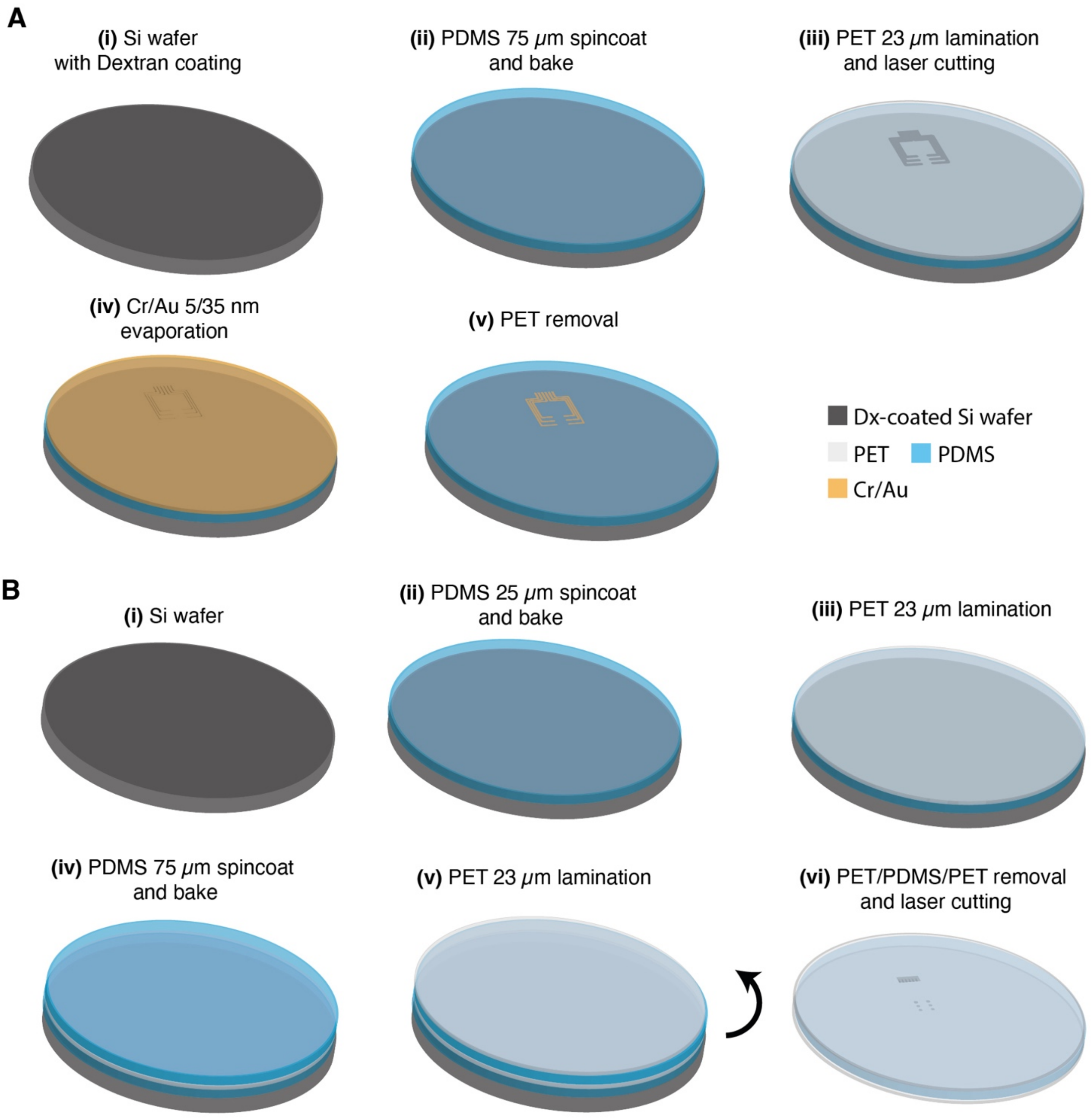

Figure S1.

Fabrication of cuff substrate (A) and encapsulation (B), with material legend.

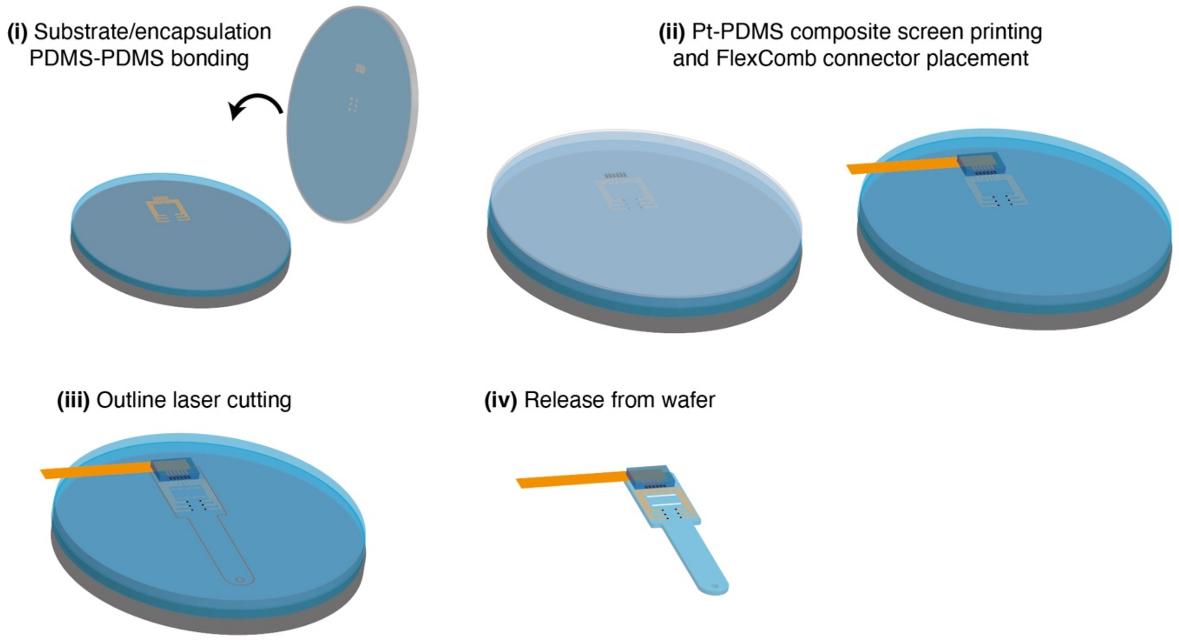

Figure S2.

Final cuff assembly, including bonding, connector integration and device release.

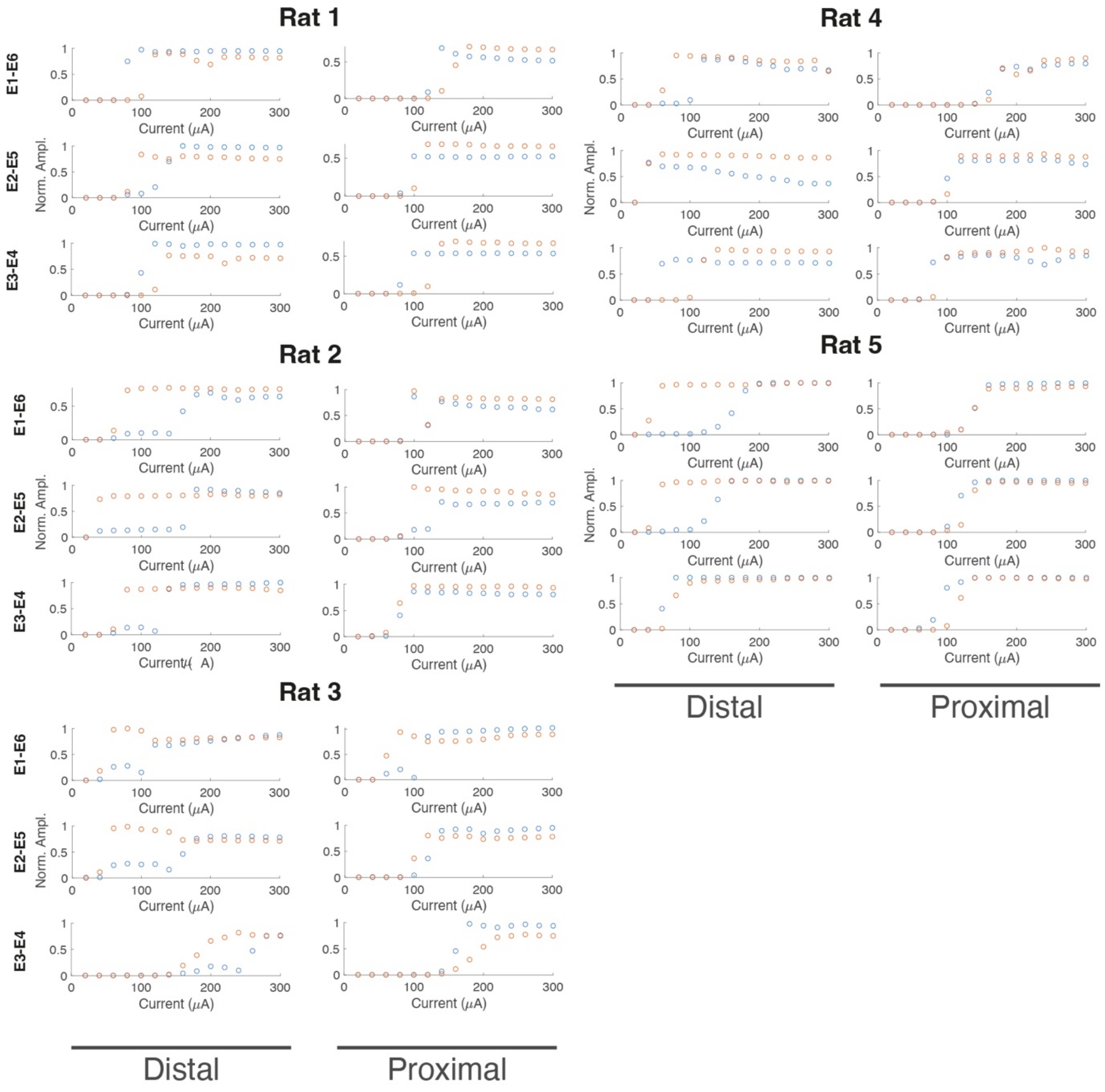

Figure S3.

Normalized EMG amplitude vs. stimulation current amplitude. Response for all rats, positions and electrode configurations. Blue circles indicate TA muscle, while red circles indicate GM.

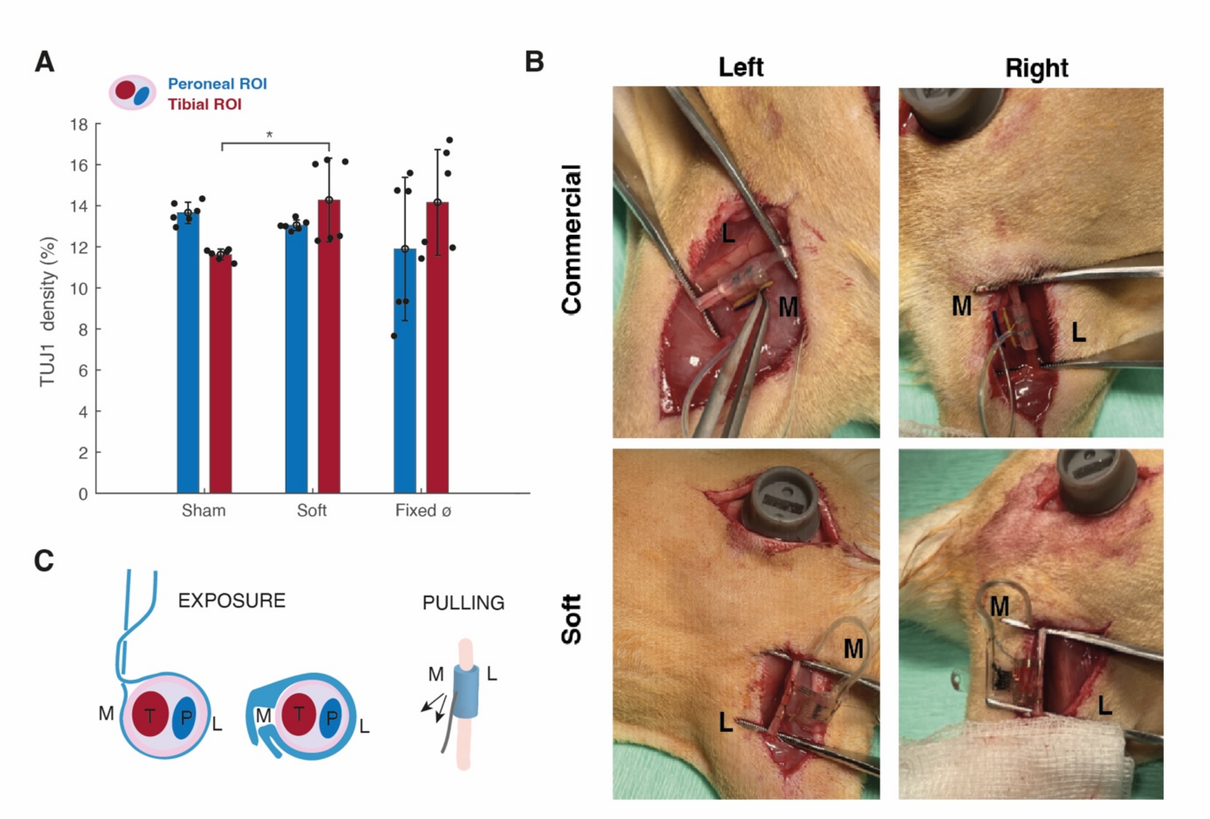

Figure S4.

Axonal density and cuff implantation. (A) Quantification of axon density in tibial and peroneal regions. Statistics: Wilcoxon rank sum test for * adjusted p<0.05, with Bonferroni correction for multiple comparisons. (B) Implantation location for all cuffs, with M and L indicating medial and lateral orientation respectively. (C) Schematic illustration of cuff and cable placement, highlighting exposed nerve side and possible pulling forces.

.

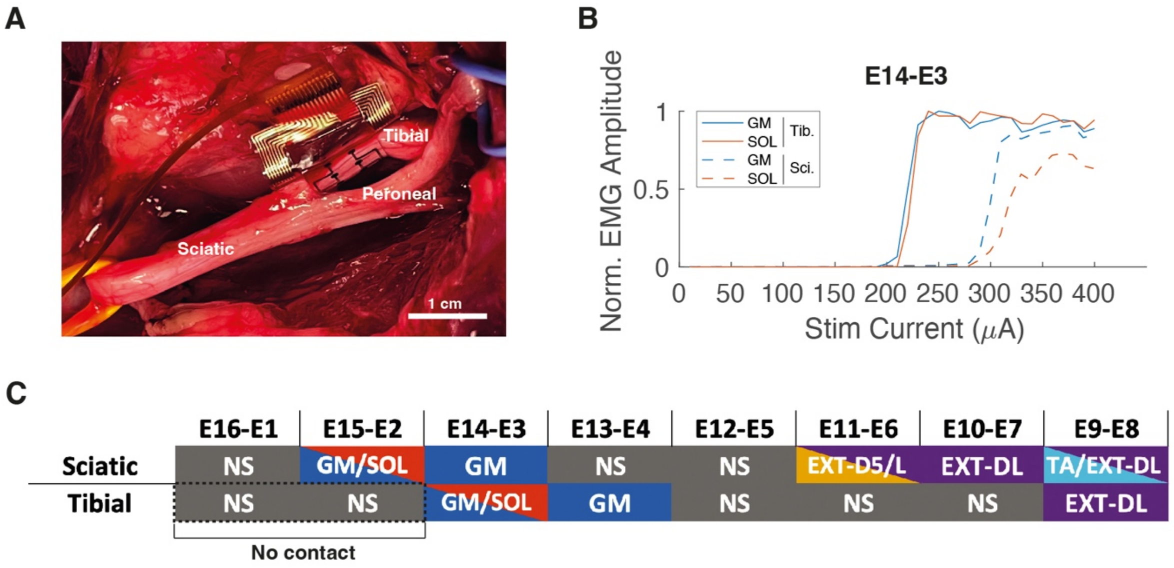

Figure S5.

Stimulation of pig tibial nerve. (A) Soft cuff implantation on tibial branch. (B) Comparison of GM and SOL activation during sciatic and tibial nerve stimulation from electrode pair E14-E3. (C) Summary table of selective muscle activation (SI>0.4, EMG>30%) in both sciatic and tibial nerve stimulation.

References

Bankhead P, Loughrey MB, Fernández JA, Dombrowski Y, McArt DG, Dunne PD, et al. QuPath: Open source software for digital pathology image analysis. Sci Rep. 2017 Dec 4;7(1):16878.

Fallegger F, Schiavone G, Pirondini E, Wagner FB, Vachicouras N, Serex L, et al. MRI-Compatible and Conformal Electrocorticography Grids for Translational Research. Advanced Science. 2021 May;8(9):2003761.

Gao Y, Wu K, Suo Z. Photodetachable Adhesion. Adv Mater. 2019 Feb;31(6):1806948.

Minev IR, Wenger N, Courtine G, Lacour SP. Research Update: Platinum-elastomer mesocomposite as neural electrode coating. APL Materials. 2015 Jan 1;3(1):014701.
